# An Empirical Evaluation of Lightweight Random Walk Based Routing Protocol in Duty Cycle Aware Wireless Sensor Networks

**DOI:** 10.1155/2014/946249

**Published:** 2014-02-13

**Authors:** Adnan Noor Mian, Mehwish Fatima, Raees Khan, Ravi Prakash

**Affiliations:** ^1^Department of Computer Science, National University of Computer and Emerging Sciences, Lahore 54700, Pakistan; ^2^Department of Computer Science, University of Texas at Dallas, Dallas, TX 75080-3021, USA

## Abstract

Energy efficiency is an important design paradigm in Wireless Sensor Networks (WSNs) and its consumption in dynamic environment is even more critical. Duty cycling of sensor nodes is used to address the energy consumption problem. However, along with advantages, duty cycle aware networks introduce some complexities like synchronization and latency. Due to their inherent characteristics, many traditional routing protocols show low performance in densely deployed WSNs with duty cycle awareness, when sensor nodes are supposed to have high mobility. In this paper we first present a three messages exchange Lightweight Random Walk Routing (LRWR) protocol and then evaluate its performance in WSNs for routing low data rate packets. Through NS-2 based simulations, we examine the LRWR protocol by comparing it with DYMO, a widely used WSN protocol, in both static and dynamic environments with varying duty cycles, assuming the standard IEEE 802.15.4 in lower layers. Results for the three metrics, that is, reliability, end-to-end delay, and energy consumption, show that LRWR protocol outperforms DYMO in scalability, mobility, and robustness, showing this protocol as a suitable choice in low duty cycle and dense WSNs.

## 1. Introduction

Wireless Sensor Networks (WSNs) are one of the emerging technologies that are making revolutionary developments in everyday scenarios. Their acceptance is rapidly increasing for a wide range of real life applications. Many applications like medical technologies, environment and habitat monitoring, law enforcement, and security are shifting their paradigm from manual system to automation by means of WSN [1, 2].

In WSN, small sensor motes are used to collect the data by means of sensing from their surrounding and the data is forwarded to a common repository or some dedicated device called sink. Sensor nodes are limited in resources and are mostly battery powered for which replacement is not realistic especially in remote applications. Generally WSNs have various design and implementation issues regarding their nature, but energy efficiency is the main design paradigm due to the limited source of energy available in sensor motes.

A sensor node can be a direct neighbor of sink or it can be a distant device out of the direct communication range of sink. In the later case, sensor nodes cooperate with each other in a multithop fashion to forward the data sensed by them and their neighbors to the sink nodes. WSNs are typically used in static environment; for example, in a factory for automated monitoring of processes in different systems, the low cost wireless nodes network would be preferred over wired sensing devices. Recently some new applications are emerging that are inherently dynamic in nature, for example, military surveillance, tracking of an object that changes its position [1], sensor nodes used in sea like dynamic water environments [2], and cattle farming. Besides generating data for its sensing activity, the repeated involvement of energy constrained sensor nodes, in forwarding packets coming from other nodes, causes its life time to finish soon. Moreover, the applications which need frequent mobility of sensor nodes can add to make this situation worse.

Deploying sensor in static and dynamic environments has its own advantages and disadvantages. Static sensors are easy to deploy and manage but can result in issues like uneven energy depletion of sensor nodes. For example, sensor nodes near sink consume more energy as most of the traffic routed to sink passes from these nodes thus resulting in shorter battery life time of these nodes. Dynamic wireless sensor environments, on the other hand, are complex in designing and deployment but have some advantages over static scenarios. Mobility allows sensor nodes to cover more area as compared to other static nodes and mobility policies can be made for taking account of energy saving. So if we allow mobility in sensor nodes while the sink is static, sensor nodes may change their geographical location. The traffic burden is shared and distributed among nodes. Similarly if we allow sinks to be mobile, it allows energy saving in whole network by changing its position according to the energy consumed by its neighbors [3].

Duty cycling is a technique in which different sensor nodes use power saving inactive mode, that is, sleep mode at different time intervals [4]. In many applications of WSNs, packets are generated periodically at low rate and then routed to sink node or sink may send different queries to different sensor nodes. Duty cycling is very suitable in low data rate scenarios where nodes get involved in activity only at specific intervals of time.

There are different routing protocols for wireless networks at network layer. These can be classified generally in broadcast and unicast based protocols. For example, flooding, gossiping, and opportunistic routing are broadcast based protocols whereas AODV, DSR, AOMDV, and DYMO are unicast protocols [5, 6]. Generally broadcast protocols show poor performance due to excessive packets generation and collisions. For these reasons, unicast protocols are preferred. In such protocols duty cycling can be used to conserve energy.

Unicast based routing protocols can be proactive, reactive, hybrid, and probabilistic by nature. Proactive (table driven) protocols use routing tables for estimating the connection between source and destination node. Reactive (on-demand) protocols do not require any precalculation and estimation for route and hence these are much appealing in dynamic environments. Hybrid protocols are usually combination of proactive and reactive approaches and tend to adapt their behavior according to network changes. Probabilistic protocols also do not require any precalculation of routes and probabilistic formulation is made for selecting the next forwarding node, thus avoiding large overheads of forming routing tables. Due to these features, probabilistic protocols are useful especially for low data rate and in dense deployment of WSN.

Random walk based routing is a probabilistic protocol in which each node selects randomly from its neighbors nodes to forward the data packet. The path thus formed is a random walk (RW). RW has been widely studied; for instance, see [7, 8] for a quick overview. RW based routing protocol is often proposed for very small devices, in large and dynamic networks due to being extremely simple to implement, requiring small memory footprints, and not requiring topology information of the network and load balancing property of the RW [9–11]. On the other hand, reactive (on-demand) routing protocols are also considered to be useful in resource constrained and dynamic WSNs. However due to their inherent properties, increasing density of nodes badly affects performance of such protocols in terms of scalability. Furthermore, high mobility of sensor nodes and enabling low duty cycling make routing quite challenging. In such scenarios, random walk based routing has not been studied widely. In this paper, we have put before a Lightweight Random Walk based Routing (LRWR) protocol in which each step follows a three messages exchange not only to discover neighbors but also to randomly select and forward the packets to the selected neighbor. We call this protocol lightweight since the number of messages required to achieve one step of RW are bare minimum. We applied the LRWR protocol in WSN with IEEE 802.15.4 standard and duty cycle enabled environments. By comparing its performance for low data rate with DYMO, a widely used protocol for WSN, we find that LRWR protocol offers a better alternative for duty cycle enabled mobile WSNs.

Rest of the paper is structured as follows.  Section 2 gives a brief overview of IEEE 802.15.4 and implementation of duty cycle in it.  Section 3 briefly describes some of the important related works.  Section 4 briefly describes the LRWR protocol.  Section 5 details the simulation setup, section 6 discusses the results, and finally section 7 concludes the study.

## 2. A Brief Overview of IEEE 802.15.4 Protocol

IEEE has established a standard 802.15.4 for low cost Low Rate Wireless Personal Area Networks (LR-WPAN) since such networks have very different requirements from other wireless networks. There are many topologies for WSN but the two underlying topologies are star and peer-to-peer.

IEEE 802.15.4 provides physical and MAC layer implementation for WSN. On physical layer it provides a low range connectivity that may vary from 10 meter to 100 meter on a data rate of 250 kbits/sec [4]. On MAC layer it provides different protocols and frame structure for implementation of low cost, low rate communication. IEEE 802.15.4 MAC can be operated on two modes either beacon enabled (synchronized) or nonbeacon enabled (unsynchronized). Nonbeacon enabled mode uses unslotted CSMA/CA while beacon enabled mode uses slotted CSMA/CA and allows duty cycling. Duty cycling is implemented with the superframe structure that consists of beacon interval and superframe duration between two beacon intervals [4].

**Figure 1:**
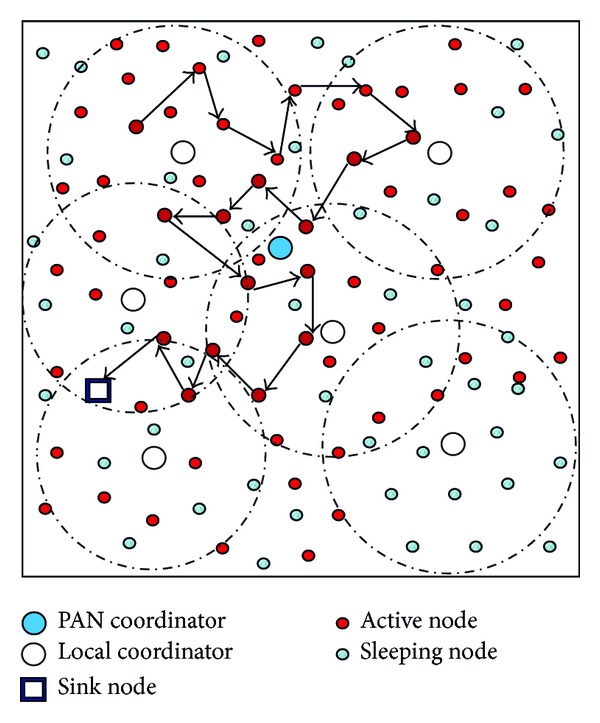
Random walk routing in a WSN with PAN Coordinators.

In beacon enabled mode, a PAN coordinator is responsible for synchronizing the nodes along with local coordinators as shown in Figure 1. These coordinators would be either synchronized or unsynchronized with respect to initialization time. If all coordinators are synchronized with respect to initialization time then nodes will have a symmetric awake and sleep behavior otherwise it will be asymmetric. In nonbeacon enabled mode, PAN coordinator is not responsible for synchronizing the PAN and due to unsynchronized environment there is no duty cycling.

Synchronization among nodes is achieved by a scanning process through all the channels for coordinator and orphan nodes. This scanning furnishes reliable synchronization; however, it increases the latency in the network. In mobile WSN, achieving synchronization is more difficult because nodes are highly inclined to change their geographical positions due to which synchronization loss occurs. In such networks latency is comparatively greater than static WSN due to synchronization loss and reconnection scans.

**Figure 2:**
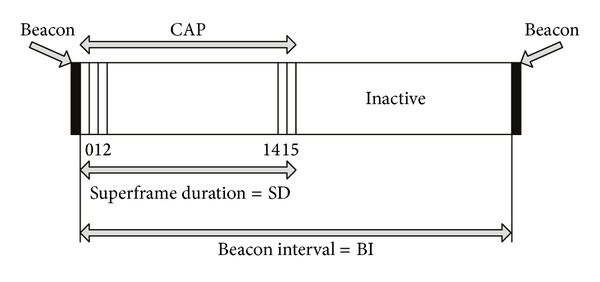
IEEE 802.15.4 superframe structure.

In IEEE 802.15.4 MAC, duty cycle is implemented by superframe structure which is the time interval between two beacons sent by coordinator as described in Figure 2. “Beacon Order” (BO) and “Superframe Order” (SO) are two parameters that are responsible for duty cycling by changing the superframe structure [4]. BO and SO are used to formulate the values of beacon interval (BI) and superframe duration (SD), respectively. BI and SD determine the active (awake) period and inactive (sleep) period for nodes that are synchronized [4]. BO and SO values can be modified explicitly. The difference of BO and SO determines the duty cycle. Different values of BO and SO can result in the same duty cycle. The duty cycle percentage according to the difference between BO and SO is given by the formulae DC = (1/2^SO-BO^)∗100 and its different values are shown in Table 1.

Duty cycling tends to increase the latency and drop ratio along with its energy saving characteristic. Although if a node has started its reception or transmission, it will go into inactive period only after completing its reception or transmission [4]. In spite of this, however, there may be packet drops due to collision of packets. These collisions may be due to ACK transmissions without backoff mechanism or due to the transmission of hidden nodes [12].

## 3. Related Work

On the network layer, there are many routing protocols that are very well known in wireless ad hoc and sensor networks. The “Dynamic MANET On-demand” (DYMO) routing protocol is intended for mobile wireless networks. It adapts to changing network topology and determines unicast routes between nodes within the network [13]. The DYMO routing protocol is successor to AODV and can work as proactive or as a reactive routing protocol [13]. There are many simulation based studies on DYMO in MANET and WSN [14–17].

Random walk based routing is unicast based protocol which has been studied extensively from different perspectives. Some of the most important related works are as follows. In [11] the authors study a scenario in which the data source sends agents that walk randomly in the network and leave traces. Sink will send queries along some other random walks and hope to encounter the data traces. In [18] based on analysis and simulations Avin and Brito study query processing in dynamic environments using RW. The paper [19] motivates the need of RW in dynamic networks and then compare the efficiency of search using flooding and biased RW in such networks. Mabrouki et al. [20] study RW based routing protocol for WSN from theoretical perspective. These works study random walk routing at an abstract level using custom built simulators assuming simplified network model. These studies do not present detail level protocols that implement random walk routing.

Ahn et al. [21] proposed an implementation of random walk protocol considering 3-way handshake, like TCP. In this protocol RW packet is forwarded to one of the neighbors which has been selected through a list of neighbors. The list of all neighbor IDs are got from a distinct neighbor discovery phase. In such a case, the RW process will be suffered significantly in case the chosen node is in sleep mode. The distinct two phases may result in multiple message exchanges that may lead to large latencies. The proposed protocol on the other hand does not have distinct two phases. Instead just in one phase of the three, both the neighbor discovery and random forwarding are achieved. This results in efficient forwarding mechanism. Moreover, [21] assumes TDMA access mechanism for communicating with nodes, which has different characteristics from CSMA used in the widely used standard IEEE 802.15.4.

The proposed protocol routes packets from source to the sink and guarantees that random walk does not branch off. The paper evaluates the performance of the protocol in duty cycle aware sensor networks and compares it with DYMO.

## 4. Lightweight Random Walk Based Routing Protocol 

**Figure 3:**
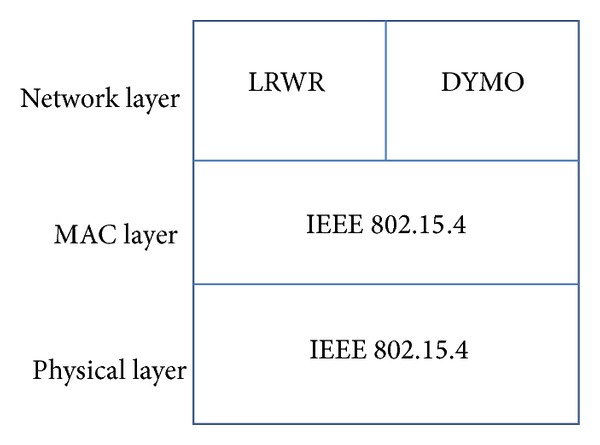
Protocol stack.

In random walk based routing the data packets are forwarded to one of the neighbors selected at random. This can be achieved in two phases. In the first phase of neighbor discovery a neighbor discovery packet is broadcasted and in return all neighbors reply with their IDs. After a specific time when all or most of the neighbors are known by the forwarding node, the node selects one of the neighbors randomly as the next hop node and then sends the packet to it. Such a simplistic approach may result in inefficiencies. Each of the phases would result in multiple message exchanges due to unicast with explicit acknowledgements and may result in large latencies due to carrier sensing mechanism. Moreover in case of dynamic environments, due to large latencies involved in neighbor discovery and then forwarding packet to one of the selected neighbors which may have moved out of the transmission range, the probability of an unexpected halting of RW increases. We present a network layer protocol, as shown in Figure 3, which not only discovers a random neighbor but also forwards the data to it in three messages exchange sequences. Due to low latencies involved in between messages, the probability of unexpected RW halting is very low.

### 4.1. Basic Assumptions

The system consists of mobile WSNs based on IEEE 802.15.4. The target node is present within the network. The data may be routed from a source sensor nodes to a sink or from sink to a sensor node. The network remains connected and there is a bidirectional connectivity between neighbor nodes at any time. The nodes can communicate only via a broadcast primitive. This primitive sends the packet using the local broadcast facility of the underlying MAC protocol, which uses the carrier sense and binary backoff mechanism to access the medium and is provided by the implementation of IEEE 802.15.4 standard.

### 4.2. Protocol Description

**Figure 4:**
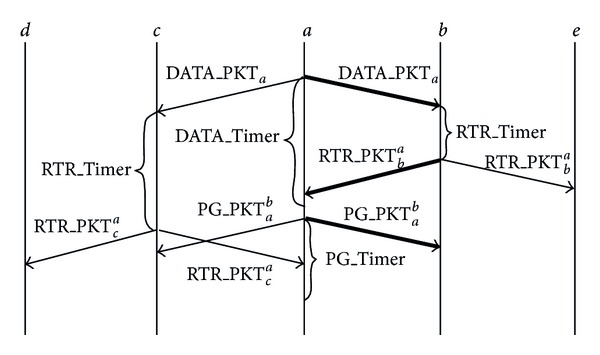
LRWR protocol sequence diagram.

The presented protocol uses three messages exchange sequence DATA/RTR/PG at network layer. The sequence diagram of the protocol is shown in Figure 4. The protocol embeds lightweight efficient distributed selection logic at the network layer as described below.

Suppose node *a* is the forwarding node that generates data packet or forwards an existing data packet DATA_PKT_*a*_ that has to be sent to some destination in the network using random walk routing. Such packets are “agents” carrying information about events or “queries” seeking such information [22]. To initiate the three messages sequence, node *a* broadcasts the packet DATA_PKT_*a*_. All nodes in the transmission range of node *a* will receive the DATA_PKT_*a*_. There is a possibility that some nodes may not receive this packet due to collisions or sleep mode. Note that it is not essential in this protocol that all of the neighbors should receive the broadcast packet. It is sufficient if some or just one of the neighbors receives it.

Now each neighbor that receives the data packet replies by broadcasting a special packet called Ready-To-Route (RTR_PKT), with a random delay RTR_Timer. This packet indicates the willingness of the neighbor node to become the new forwarding node. The random delay decreases the probability of collisions at the node *a*. If no RTR_PKT is received till the expiry of the DATA_Timer, the DATA_PKT_*a*_ would be rebroadcasted. The node *a* will accept only the first RTR_PKT and after receiving the first one, say RTR_PKT_*b*_^*a*^ packet from one of the neighbors node *b*, discards any other RTR_PKT from other neighbors. The packet RTR_PKT_*b*_^*a*^ can only be processed by node *a* and all other nodes shall discard this packet. In the Figure 4 node *e* does not process RTR_PKT_*b*_^*a*^. Similarly RTR_PKT_*c*_^*a*^ will be discarded by node *d*.

Node *a*, after accepting the request to route from node *b*, sends a special packet called Permission-Granted (PG_PKT_*a*_^*b*^). This packet is to grant permission to node *b* in response to its request to route. When other neighbor nodes receive this packet, they cancel their RTR_Timers to stop transmission of any RTR_PKT packets if not yet transmitted, thus reducing the number of unnecessary transmissions. Once node *b* gets the permission granted, it initiates its own three messages sequence by broadcasting the data packet, which now also acts as an acknowledgement for node *a* that PG packet was received at node *b*. If the forwarding node *a* does not receive DATA_PKT from the new selected forwarding node within PG_Timer expiry, the PG_PKT_*a*_^*b*^ is rebroadcasted. The newly selected forwarding node *b* only accepts the first PG_PKT_*a*_^*b*^ and discards the remaining. Now if node *b* is a destination node then neighbors do not generate any further RTR_PKT packets and the algorithm terminates otherwise the same three messages exchange sequence is repeated.

Note that in the protocol, RTR_Timer is not the only reason that results in random selection of neighbor node. Other reasons which may lead to random selection are duty cycling and random delays due to MAC layer carrier sensing.

An important aspect of this protocol is that the random walk path does not branch off. The branching occurs if more than one RTR packet from different neighbor nodes are processed, as each processed RTR packet will trigger a PG packet to a different node thus starting a different RW. This is not possible because only the first RTR is processed and all other received RTR packets are discarded. The branching can also occur if a node receiving a PG packet triggers the broadcast of a data packet, which initiates a new RW. In such a case a node that receives a PG packet, which is not part of the current hop message exchange sequence or due to multiple transmissions of PG packets from the forwarding node, will trigger the broadcast of another data packet thus causing branching. But in the proposed protocol this will not be possible because the protocol makes sure that only the first PG packet received, which is part of the current hop 3-way message exchange sequence, is processed and all others are discarded.

In Figure 4 bold lines show the interaction between the forwarding node and the neighbor node selected as a new forwarding node.

The working mechanism of the proposed protocol can be summarized as below.

- When a node wants to forward data, it broadcasts the DATA_PKT.
- All neighbor nodes receiving the DATA_PKT schedule their RTR_Timer timers randomly.
- When RTR_Timer timers (of neighbor nodes) expire, RTR_PKT packets are unicasted to the data forwarding node.
- The first RTR_PKT received on the forwarding node is accepted and all other RTR_PKT packets are discarded.
- If the forwarding node does not receive any RTR_PKT packet after waiting for a time set by DATA_Timer, it rebroadcasts the same packet. This is repeated for a specific number of times.
- The forwarding node unicast a permission granted packet PG_PKT to the neighbor node whose RTR_PKT packet was accepted. If the selected neighbor node is the destination node then algorithm terminates.
- All other neighbor nodes that receive this PG_PKT packet will halt their transmission of RTR_PKT if their RTR_timers not yet expired.
- After sending the PG_PKT, if the forwarding node does not receive DATA_PKT from the selected neighbor node after time set by PG_Timer, the PG_PKT is sent again. This is repeated for a specific number of times.
- Once a PG_PKT is received by the selected neighbor node, all other PG_PKT packets are discarded.

## 5. Simulation Setup

We did all simulation experiments using NS-2 version 2.34. We used random waypoint mobility model for generating mobility pattern of nodes and performed simulations on multiple scenario files with the same initial parameters.

In case of static scenario we randomly deployed 100, 200, 300, 400, and 500 static nodes that form cluster head tree topology in 200 m × 200 m area. All sensor nodes had transmission range of 40 meters and same initial energy. The simulations were run for 500 seconds. On application layer, a data sending application was used that sent data to a sink in the network.

Similarly in case of dynamic nodes scenario, we assumed a cluster head tree topology of 100 nodes that are mobile in 500 m × 500 m area. All nodes had transmission range of 40 meters and same initial energy. The simulations were run for 500 seconds. On application layer, similar to the static case, a data packets generating application was used that sent data to a sink node in the network. Each value of the plot is calculated as mean of 200 runs.

Both of the simulation environments were unsynchronized with respect to time. This means that even when all nodes have the same duty cycle set, some nodes may be in sleep mode. This is due to the association of nodes with different coordinators. The synchronization of nodes depends upon the device initialization time and association time with coordinator. The parameters used in the simulations are summarized in Table 2.

## 6. Results and Discussion

Our simulation stack architecture is shown in Figure 3. We compared LRWR and DYMO protocols with respect to three metrics, that is, packet delivery ratio, end-to-end delay for data delivery from source to sink node within a specific time, and energy consumption for the routes. These results are evaluated using different duty cycles and in both static and dynamic scenarios. In all of these experiments, node 0 was taken as a source node which generates data and sends it to a sink node. The results are discussed below.

### 6.1. Scalability Results

In these simulations we evaluated scalability of the protocol, that is, the performance of the protocol when the number of nodes are increased in terms of packet delivery ratio, end-to-end delay, and energy consumption in the presence of different duty cycle.

**Figure 5:**
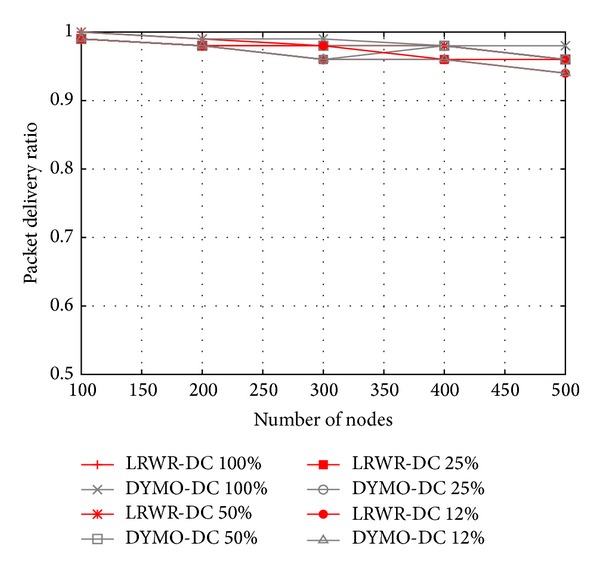
Packet delivery ratio versus total number of nodes.

Figure 5 shows the packet delivery ratio from source to sink as a function of the number of nodes. The packet delivery ratio of LRWR and DYMO is similar even with low duty cycle. Since nodes are static in the network, therefore duty cycling has very less effect on delivery ratio. However, with the increase in network density, there is a little decline in delivery ratio in both of the protocols.

**Figure 6:**
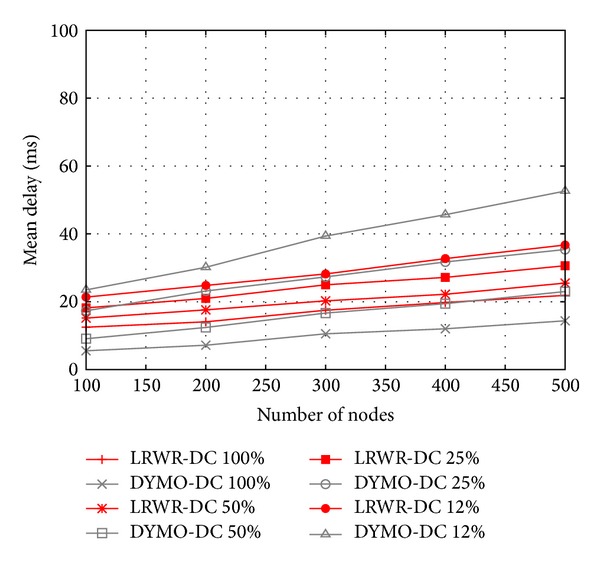
Mean delay versus total number of nodes.

Figure 6 shows the mean end-to-end delay from source to sink as a function of number of nodes in both LRWR and DYMO protocols for different duty cycles in static nodes. We see that the mean delay increases with increasing number of nodes. This is quite initiative since with the increase of number of nodes the number of hops in the path from source to sink increases. We also see that the mean delay increases with decreasing duty cycle. This is because in low duty cycle more nodes are inactive and forwarding of packets is delayed. Interestingly the mean delay in very low duty cycles increases at a faster rate in DYMO as compared with LRWR with increasing number of nodes. This is because in DYMO whenever a node is not approachable its entry is deleted from the forwarding node routing table and then a route discovery process is initiated again causing high delay. On the other hand LRWR immediately selects randomly any active node and forwards the packet to it resulting in comparatively low delays. Thus LRWR protocol performance with respect to mean end-to-end delay is better as compared with DYMO in low duty cycles and large number of nodes.

**Figure 7:**
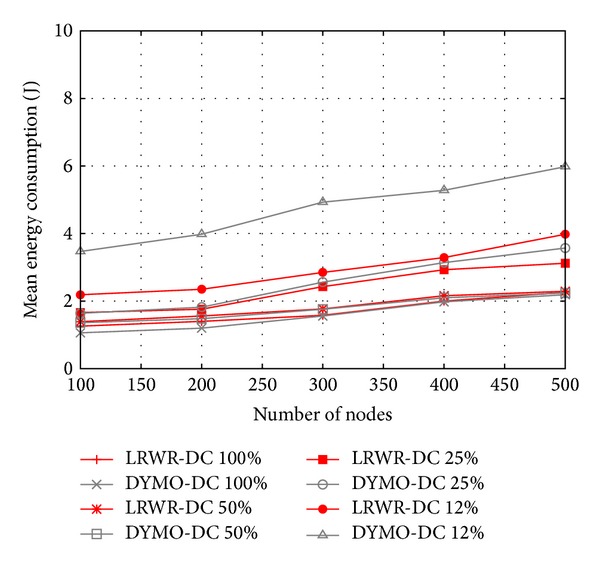
Mean energy consumed versus total number of nodes.

In Figure 7 mean energy consumption of nodes is shown as a function of the number of nodes. These results of LRWR and DYMO are evaluated under different duty cycles. Generally the energy consumption is high because more effort is required to achieve synchronization between coordinators and sensor nodes [23]. From the plots, we see in both protocols that with the increase in the number of nodes, the energy consumption also increases. This is because of the increase in the number of hops and thus transmissions. However it is observed that the energy consumption of LRWR is less than that of DYMO in low duty cycles. In DYMO routes from source to sink need to be discovered and in case of low duty cycles, since more nodes may be in sleep mode, more effort is required to find routes to sink.

### 6.2. Mobility Results

In this section we discuss the results of mobility scenario and compare both protocols in the presence of mobility. In the experiments we executed a data generation application that sent data from the source to sink node. We deployed 100 nodes. Node 0 was taken as source and node 99 was taken as sink node in all the experiments. We compared the results of both LRWR and DYMO protocols using three metrics, namely, packet delivery ratio, end-to-end delay for data delivery within a specific time, and energy consumption for the route.

**Figure 8:**
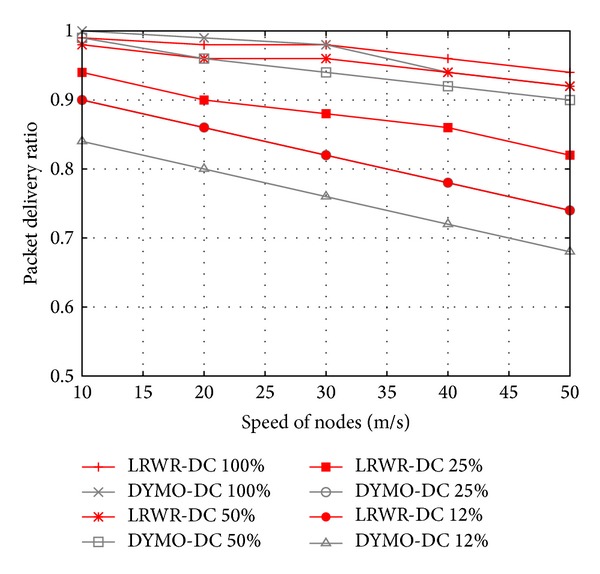
Packet delivery ratio versus speed of nodes.

Figure 8 shows the packet delivery ratio as a function of the speed of nodes. These results of LRWR and DYMO are evaluated using different duty cycles. The packet delivery ratios of LRWR and DYMO are similar at high duty cycle. Since nodes are mobile in the network, therefore, with the increase in speed of nodes, data delivery ratio decreases. The reason of this decline is that mobile nodes tend to lose connectivity and synchronization with the coordinators more often due to their change of geographical position and having to reassociate themselves with some coordinators. Due to this synchronization loss and reassociation, packet loss is more often. DYMO is deterministic protocol that maintains the route on-demand. These routes are fixed and packet delivery failure would be due to packet loss and unassociation of some nodes (called ophran nodes). While LRWR is a probabilistic protocol that finds the next forwarding node among the active neighbors and if some nodes are off or unsynchronized, these will not be considered in next forwarding node selection. Due to the similar reasons the low duty cycles also drastically affect DYMO as compared with LRWR protocol. In short we find that the proposed LRWR protocol performance is better in low duty cycles and high mobility.

**Figure 9:**
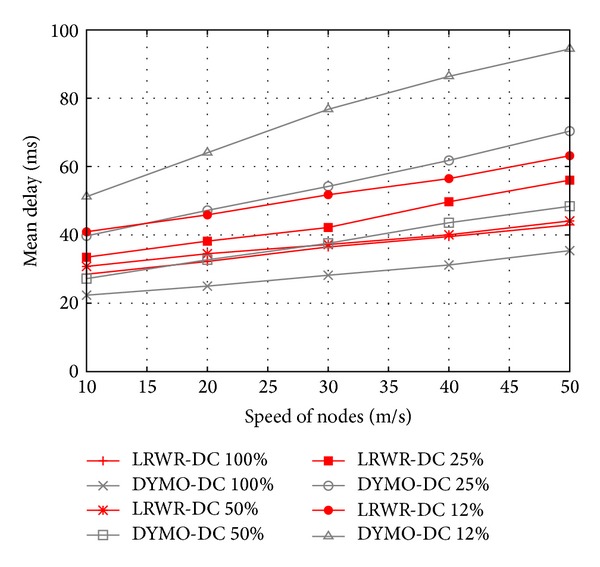
Mean delay versus speed of nodes.

Figure 9 shows the mean delay for data delivery as a function of the speed of nodes. These results of LRWR and DYMO are evaluated under different duty cycles. At 100% duty cycle with mobile nodes DYMO performs better than LRWR. At 50% duty cycle with mobile nodes LRWR protocol mean delay is similar to DYMO. However in low duty cycles 25% and 12% and with high speed of nodes LRWR is performing better than DYMO. The reason of this performance decline of DYMO is that mobile nodes tend to loose connectivity with coordinators more often, due to their change of geographical position. This causes loss of synchronization in the network. With the increase of speed, synchronization loss is even more often. Due to the deterministic nature and routing table based, DYMO has to forward packets according to its routing information. It might be possible that in a specific route some nodes are in sleep mode. In such a case a node has to wait for the next node to go into active mode. This increases latency. LRWR, on the other hand, takes the advantage of probabilistic nature and randomly selects one of the available active neighbors.

**Figure 10:**
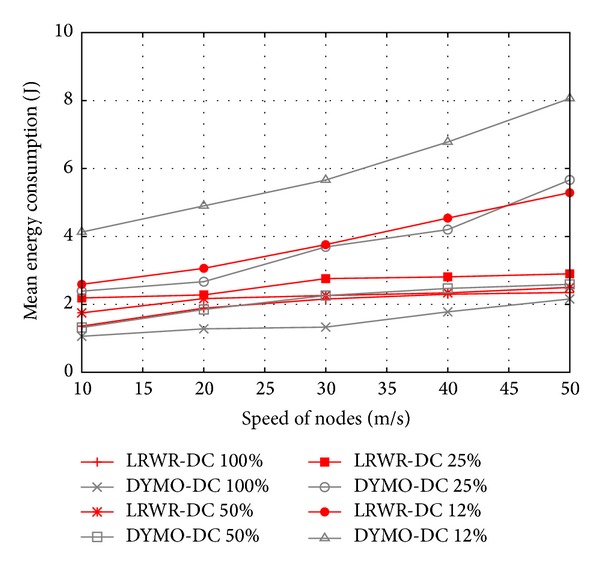
Mean energy consumed versus speed of nodes.

In Figure 10 mean energy consumption of nodes is shown as a function of the speed of nodes in the presence of different duty cycles. We see that in low duty cycles and high mobility LRWR protocol performs better than DYMO. The DYMO consumes more energy since it has to generate more control packets to maintain routes and synchronization in higher mobility and lower duty cycle. LRWR protocol, on the other hand, simply forwards the data packets to any of the available active nodes without having an overhead of maintaining routes and synchronization.

### 6.3. Stability Results

**Figure 11:**
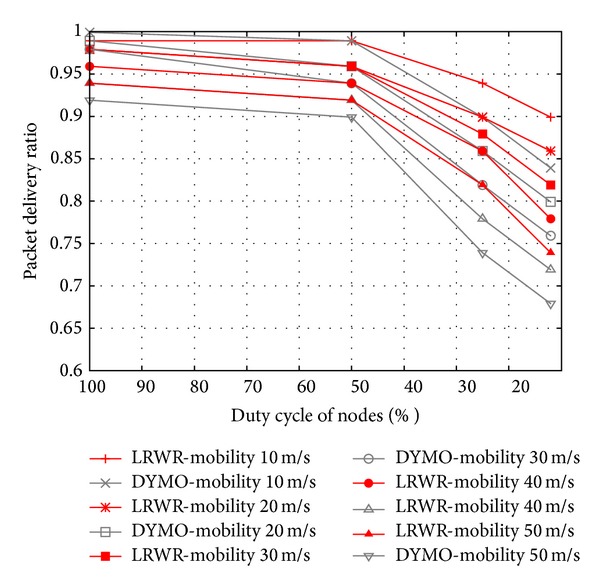
Packet delivery ratio versus duty cycle of nodes.

In this experiment we have compared the performance of both of the protocols in terms of packet delivery ratio under decreasing duty cycles with different speeds. In Figure 11 LRWR protocol shows more robust and stable behavior in duty cycle aware WSN. By reducing duty cycle of nodes, DYMO gets steep decline in packets delivery ratio whereas LRWR shows less decrease in its delivery ratio. When duty cycle is decreased, though the number of active nodes gets lesser, LRWR protocol still maintains its high data delivery ratio. This higher delivery ratio even with the support of less number of nodes shows the robustness of LRWR protocol.

## 7. Conclusion and Future Work

In this paper, we have presented a network layer unicast three-way messages exchange protocol LRWR and analyzed its performance based on packet delivery ratio, end-to-end delay, and energy consumption in the presence of duty cycles.

Based on extensive simulations of the proposed protocol with IEEE 802.15.4 lower layers, we found that the proposed LRWR protocol outperforms DYMO protocol especially in low duty cycles and in large number of nodes. We did simulation experiments for both static and dynamic environments. From the static simulation results, we find the scalability of both protocols. We see that the packet delivery ratio scale well in both protocols. However the mean energy and delay are higher in case of DYMO as the number of nodes increases and the duty cycle is reduced. From the dynamic environment experiments we observe that the packet delivery ratio decreases in both cases. LRWR performs better in high mobility and in low duty cycles. Mean delay and energy consumed increase in both cases but the increase is greater in case of DYMO with the increase of speed of nodes. Thus LRWR performs better than DYMO even under high mobility.

In this paper we studied network in synchronized environments. Synchronization has an overhead of the synchronization protocol which affects adversely. It would be interesting to see the effects when the network is unsynchronized and duty cycling is obtained at node level rather than at coordinator level. We plan to extend our experiments for such unsynchronized mobile WSN in the future.

## Figures and Tables

**Table 1:** Variation in duty cycle with BO and SO values [4].

BO-SO	0	1	2	3	4	5
DC	100	50	25	12	6.25	3.125
BO-SO	6	7	8	9	>10	—
DC	1.56	0.78	0.39	0.195	<0.1	—

**Table 2:** Simulation parameters.

Parameter	Scenario	
	Static	Mobile
Simulation time	500 sec	500 sec
Area	200 m × 200 m	500 m × 500 m
Number of nodes	100, 200, 300, 400, 500	100
Trans. range	40 m	40 m
Duty cycle	100%, 50%, 25%, 12%	100%, 50%, 25%, 12%
PAN coordinator	1	1
Local coordinators	39, 78, 117, 156, 195	39
Mobility model	Random deployment	Random waypoint
Speed of nodes	0 m/s	10, 20, 30, 40, 50 m/s
Pause time	—	2 sec
